# Consolidation chemotherapy may improve survival for patients with locally advanced non-small-cell lung cancer receiving concurrent chemoradiotherapy - retrospective analysis of 203 cases

**DOI:** 10.1186/s12885-015-1710-2

**Published:** 2015-10-16

**Authors:** Lipin Liu, Nan Bi, Zhe Ji, Junling Li, Jingbo Wang, Xiaozhen Wang, Zhouguang Hui, Jima Lv, Jun Liang, Zongmei Zhou, Yan Wang, Weibo Yin, Luhua Wang

**Affiliations:** 1 Department of Radiation Oncology, Cancer Hospital and Institute, Chinese Academy of Medical Sciences & Peking Union Medical College, Beijing, 100021 China; 2 Department of Medical Oncology, Cancer Hospital and Institute, Chinese Academy of Medical Sciences & Peking Union Medical College, Beijing, 100021 China

**Keywords:** Locally advanced, Non-small-cell lung cancer, Consolidation chemotherapy, Efficacy, Toxicity

## Abstract

**Background:**

For patients with locally advanced non-small-cell lung cancer (LA-NSCLC), the role of consolidation chemotherapy (CCT) following concurrent chemoradiotherapy (CRT) is partially defined. The aim of this study was to evaluate the efficacy and toxicity of CCT.

**Methods:**

The characteristics of LA-NSCLC patients treated with curative concurrent CRT from 2001 to 2010 were retrospectively reviewed.

**Results:**

Among 203 patients, 113 (55.7 %) patients received CCT. The median number of delivered CCT was 3 and 89.4 % patients completed ≥2 cycles. The OS was significantly better for patients in the CCT group compared with that in the non-CCT group (median OS, 27 months vs. 16 months; 5-year OS, 30.4 % vs. 22.5 %; *p* = 0.012). The median PFS were 12 months in the CCT group and 9 months in the non-CCT group (*p* = 0.291). The survival advantages of CCT were significant for males (HR: 0.63; 95 % CI, 0.44 − 0.90), patients with age < 60 years (HR: 0.63; 95 % CI, 0.42 − 0.95), non-squamous histology (HR: 0.44; 95 % CI, 0.25 − 0.76), pretreatment KPS ≥ 80 (HR: 0.67; 95 % CI, 0.48 − 0.93), stage IIIb (HR: 0.64; 95 % CI, 0.43 − 0.95), stable disease (HR: 0.31; 95 % CI, 0.14 − 0.65) and radiotherapy dose ≥ 60 Gy (HR: 0.69; 95 % CI, 0.48 − 1.00). There was no significant difference between the CCT group and the non-CCT group regarding treatment-related toxicities.

**Conclusions:**

CCT might further prolong survival compared with CRT alone for LA-NSCLC without increasing treatment-related toxicities, especially for males, patients with age < 60 years, non-squamous histology, pretreatment KPS ≥ 80, stage IIIb, stable disease and radiotherapy dose ≥ 60 Gy. Large size prospective investigations that incorporate patient characteristics and treatment response are warranted to validate our findings.

**Electronic supplementary material:**

The online version of this article (doi:10.1186/s12885-015-1710-2) contains supplementary material, which is available to authorized users.

## Background

Lung cancer remains the leading cause of cancer-related deaths worldwide [1]. Non-small-cell lung cancer (NSCLC) accounts for 80 % of all lung cancer cases and approximately 40 % of patients with NSCLC present with locally advanced non-small-cell lung cancer (LA-NSCLC) at diagnosis [2]. The standard-of-care treatment for LA-NSCLC is concurrent platinum-based chemotherapy and thoracic radiotherapy [3–5], which yields superior survival compared with either radiotherapy alone or sequential chemoradiotherapy. However, the outcome of LA-NSCLC treated with concurrent chemoradiotherapy (CRT) remains disappointing, with a median survival of 12–23.2 months [6, 7].

To improve survival, numerous studies have focused on exploring the feasibility and efficacy of consolidation chemotherapy (CCT) following concurrent CRT with discordant results. A phase II study of the Southwest Oncology Group (SWOG) 9504 [8] treated patients with concurrent CRT followed by consolidation docetaxel and achieved a promising median survival of 26 months suggesting a possible benefit of CCT. However, the Hoosier Oncology Group (HOG) [6], who published the only full article on a randomized phase III trial thus far, failed to replicate the encouraging outcome of SWOG 9504 by randomly delivering either docetaxel or observation after CRT. A recent pooled analysis [2] of 45 studies showed that CCT provided no survival benefit for LA-NSCLC patients. However, a subgroup analysis demonstrated that Asian populations (mostly from Japan and Korea) tended to benefit from CCT, although this benefit did not meet statistical significance (HR = 0.84; 95 % CI, 0.68-1.04; *p* = 0.105). Given the lack of substantial evidence from randomized phase III clinical trials, the definitive role of CCT in LA-NSCLC is unknown, especially in the Asian population. Therefore, our study attempted to evaluate the efficacy and toxicity of CCT after concurrent CRT at our institution.

## Methods

### Ethics statement

This retrospective study was approved by the ethics committee of the Cancer Hospital and Institute of Chinese Academy of Medical Sciences & Peking Union Medical College. Informed consent was exempted by the board due to the retrospective nature of this research. Patient records were anonymized and de-identified prior to analysis.

### Eligibility

We retrospectively reviewed the clinical records of LA-NSCLC patients treated with concurrent CRT as an initial treatment at out institution between January 2001 and December 2010. The criteria for inclusion were defined as follows: (1) histologically or cytologically proven NSCLC; (2) clinically diagnosed as stage III disease according to the American Joint Committee on Cancer (AJCC) 2009 staging system; (3) treated with curative thoracic radiotherapy of no less than 50 Gy using intensity modulated radiotherapy (IMRT) or three-dimensional conformal radiotherapy (3D-CRT) with concurrent platinum doublet chemotherapy; (4) treatment responses evaluated 1 month after the completion of concurrent CRT in accordance with the Response Evaluation Criteria for Solid Tumors (RECIST) version 1.1 as complete response (CR), partial response (PR), and stable disease (SD).

### Evaluation and follow-up

Complete blood cell counts (CBCs) and blood chemistry examinations were repeated once per week during the treatment period. The follow-up evaluations consisted of a physical examination, CBC, serum biochemistry, tumor marker, thoracic computed tomography (CT) scans, abdomen B-ultrasound examination, and other necessary imaging examinations as clinically indicated at intervals of 3 months for the first year, then every 6 months for the following 2 years, and annually thereafter. Local recurrence was defined as primary tumor recurrence, and regional recurrence was defined as recurrence in the mediastinum, hilum and supraclavicular fossa. Other sites of recurrence, including contralateral lung and metastatic lymph nodes in the neck or axilla, were defined as distant metastasis. Disease progression was determined based on a radiologic examination, histologic examination, or both. Treatment toxicities were graded according to the Common Terminology Criteria for Adverse Events (CTCAE) version 3.0.

### Data analysis

Overall survival (OS) was defined from the beginning of concurrent CRT to the time of death due to any cause or last follow-up. Cancer specific survival (CSS) was defined from the beginning of concurrent CRT to the time of death due to lung cancer or last follow-up. Progression-free survival (PFS) was defined from the beginning of concurrent CRT to the time of tumor progression or last follow-up. Local regional progression-free survival (LRPFS) was defined from the beginning of concurrent CRT to the time of local regional progression or last follow-up. Distant metastasis-free survival (DMFS) was defined from the beginning of concurrent CRT to the time of appearance of metastatic disease or last follow-up. Survival analysis was performed using the Kaplan-Meier method and log-rank test. Univariate and multivariate analyses by use of a Cox-proportional hazards model were performed to evaluate potential prognostic factors for OS and PFS. Variables with *p* < 0.3 in univariate analyses were entered into multivariate analyses. The Pearson *χ*^2^ test was used to compare the baseline characteristics and incidence of specific toxicities between treatment groups. Cox proportional hazards models, stratified by age, sex, histology, pretreatment Karnofsky performance score (KPS), stage, treatment response and radiotherapy dose were used to estimate HRs and 95 % confidence intervals (CIs) and test for significance for OS. A statistically significant difference was defined as *p* < 0.05. All data were processed with SPSS version 19.0.

## Results

### Patient characteristics

This retrospective study identified 261 consecutive LA-NSCLC patients who received concurrent chemotherapy and curative thoracic radiotherapy with a radiation dose ≥ 50 Gy at our institution between January 2001 and December 2010. We excluded 17 patients whose response assessments were unavailable, 13 patients who experienced disease progression within a month after concurrent CRT, 18 patients whose concurrent chemotherapy did not consist of platinum doublet regimens and 10 patients who were treated with conventional two-dimensional radiotherapy; thus, a total of 203 patients were available for analysis. The characteristics of the 203 patients are presented in Table 1. The median follow-up time was 23 months (range, 2–130 months) for the entire study population and 58.5 months (range, 10–130 months) for censored patients. The median age of the patients was 56 years (range, 31–73 years). The majority of patients were male (83.7 %) and younger than 60 years old (64 %) with no significant (<5 %) weight loss (82 %) and a smoking index > 400 (60.6 %). 94.6 % of patients had a pretreatment KPS ≥ 80, and 66.5 % of patients presented with stage IIIb disease. Most patients had normal hemoglobin (95.6 %) and carcinoembryonic antigen (CEA) (67.1 %) levels at diagnosis. The most common histology subtype was squamous cell carcinoma (SCC) (65.5 %). Only 26.1 % of patients had positron emission tomography (PET) scan staging.

**Table 1:** Patient characteristics

Characteristic	Non-CCT (%)	CCT (%)	*p*-value
Gender			
Male	83 (92.2)	87 (77.0)	0.003
Female	7 (7.8)	26 (23.0)	
Age			0.005
< 60 years	48 (53.3)	82 (72.6)	
≥ 60 years	42 (46.7)	31 (27.4)	
Weight loss			0.941
< 5 %	74 (82.2)	90 (81.8)	
≥ 5 %	16 (17.8)	20 (18.2)	
Smoking index^a^			0.031
≤ 400	28 (31.1)	52 (46.0)	
> 400	62 (68.9)	61 (54.0)	
Pretreatment hemoglobin			0.306
< 120 g/L	2 (2.2)	7 (6.2)	
≥ 120 g/L	88 (97.8)	106 (93.8)	
Pretreatment KPS			0.697
< 80	6 (6.7)	5 (4.4)	
≥ 80	84 (93.3)	108 (95.6)	
Stage			0.146
IIIa	35 (38.9)	33 (29.2)	
IIIb	55 (61.1)	80 (70.8)	
Histology subtype			0.545
SCC	61 (67.8)	72 (63.7)	
Non-SCC	29 (32.2)	41 (36.3)	
Pretreatment CEA			0.729
< 5 ng/ml	50 (68.5)	62 (66.0)	
≥ 5 ng/ml	23 (31.5)	32 (34.0)	
PET scan staging			
Yes	25 (27.8)	28 (24.8)	0.629
No	65 (72.2)	85 (75.2)	
Radiotherapy technique			0.010
3D-CRT	26 (28.9)	16 (14.2)	
IMRT	64 (71.1)	97 (85.8)	
Radiotherapy dose			0.342
≥ 60 Gy	71 (78.9)	95 (84.1)	
< 60 Gy	19 (21.1)	18 (15.9)	
Concurrent chemotherapy			0.010
EP	36 (40.0)	63 (55.8)	
PC	49 (54.4)	38 (33.6)	
others	5 (5.6)	12 (10.6)	
Response			0.559
CR + PR	72 (80.0)	94 (83.2)	
SD	18 (20.0)	19 (16.8)	

*CCT* consolidation chemotherapy, *CEA* carcinoembryonic antigen, *KPS* Karnofsky performance status, *PET* positron emission tomography, *SCC* squamous cell carcinoma

^a^Smoking index is the number of cigarettes smoked per day × the number of cigarette-years

Of all 203 patients, 161 (79.3 %) were treated with IMRT and 42 (20.7 %) were treated with 3D-CRT. The radiation area only included the involved fields. The median radiation dose was 60 Gy in 30 fractions (range, 50–74 Gy in 25–37 fractions). For the concurrent chemotherapy regimen, 99 (48.8 %) patients were administered EP (etoposide plus cisplatin), 87 (42.8 %) patients received PC (paclitaxel plus carboplatin) and 17 (8.4 %) patients were treated with other platinum-doublet regimens. The responses of CR, PR, and SD were observed in 5 (2.5 %) patients, 161 (79.3 %) patients and 37 (18.2 %) patients, respectively. After concurrent CRT, 113 (55.7 %) patients received CCT, including 88 patients with platinum-based doublet chemotherapy regimens, and 25 patients with single-agent regimens. Among 113 patients who received CCT, the median number of delivered CCT was 3 and 101 (89.4 %) patients completed ≥2 cycles of CCT.

As shown in Table 1, females (23 % vs. 7.8 %; *p* = 0.003), patients aged < 60 years (72.6 % vs. 53.3 %; *p* = 0.005) with a smoking index ≤ 400 (46 % vs. 31.1 %; *p* = 0.031) who received IMRT (85.8 % vs. 71.1 %; *p* = 0.010) and concurrent EP chemotherapy (55.8 % vs. 40 %; *p* = 0.010) were more prevalent in the CCT group than in the non-CCT group. The remaining listed clinical characteristics were comparable between the two groups.

### Survival and prognostic factors

**Fig. 1:**
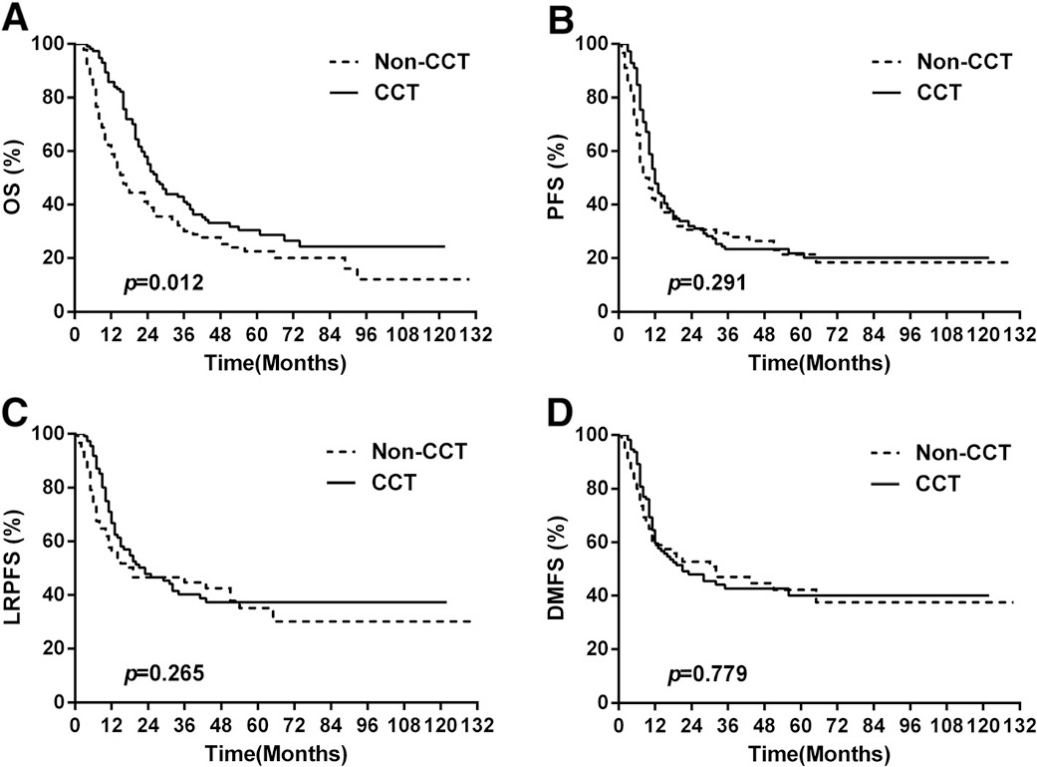
Comparison of **a** overall survival (OS), **b** progression-free survival (PFS), **c** local regional progression-free survival (LRPFS) and **d** distant metastasis-free survival (DMFS) between the consolidation chemotherapy (CCT) and non-CCT groups

The median OS and 5-year OS for all patients were 24 months and 26.9 %, respectively. Patients in the CCT group achieved significant survival prolongation compared with those in the non-CCT group (median OS, 27 months vs. 16 months; 5-year OS, 30.4 % vs. 22.5 %; *p* = 0.012; Fig. 1a). The median CSS and 5-year CSS for the CCT group (28 months and 34.4 %) in our study were also superior to those for the non-CCT group (17 months and 27.9 %) (*p* = 0.022), which was consistent with the OS results. The median PFS and 5-year PFS were 12 months and 21.8 % in the CCT group and 9 months and 21.4 % in the non-CCT group, respectively (*p* = 0.291; Fig. 1b). The 5-year LRPFS were 37.3 % in the CCT group and 35.1 % in the non-CCT group (*p* = 0.265; Fig. 1c). The 5-year DMFS were 40.1 % in the CCT group and 42.2 % in the non-CCT group (*p* = 0.779; Fig. 1d).

The results of the univariate and multivariate analyses of potential prognostic factors for OS are shown in Table 2. Univariate analysis identified the radiotherapy dose < 60 Gy (*p* = 0.014), no CCT (*p* = 0.012) and SD (*p* = 0.035) as significant unfavorable prognostic factors. Multivariate analysis identified pretreatment CEA ≥ 5 ng/ml (*p* = 0.047), no CCT delivery (*p* = 0.008), and SD (*p* = 0.036) as predictors for poor OS. Additional file 1: Table S1 shows the results of the univariate and multivariate analyses of potential prognostic factors for PFS. The univariate analysis showed superior PFS for patients with SCC histology (*p* = 0.013), normal pretreatment CEA (*p* = 0.000), radiotherapy dose ≥ 60 Gy (*p* = 0.019) and CR or PR (*p* = 0.049). In the multivariate analysis, age < 60 years (*p* = 0.012), pretreatment CEA ≥ 5 ng/ml (*p* = 0.000), and no CCT delivery (*p* = 0.022) were significantly associated with unfavorable PFS.

**Table 2:** Results of the univariate and multivariate analyses of prognostic factors for OS

	Univariate analysis			Multivariate analysis		
Characteristic	MST (mos)	5-yr OS (%)	*p*-value	HR	95 % CI	*p*-value
Gender			0.431			
Male	23	27.4				
Female	28	24.5				
Age			0.834			
< 60 years	24	26.5				
≥ 60 years	21	27.6				
Weight loss			0.292	1.01	0.61–1.66	0.977
< 5 %	24	28.7				
≥ 5 %	24	20.2				
Smoking index			0.399			
≤ 400	26	26.5				
> 400	20	27.4				
Pretreatment hemoglobin			0.580			
< 120 g/L	35	-				
≥ 120 g/L	24	26.8				
Pretreatment KPS			0.096	0.68	0.34–1.37	0.285
< 80	16	9.1				
≥ 80	24	28.0				
Stage			0.303			
IIIa	27	31.1				
IIIb	23	24.9				
Histology subtype			0.848			
SCC	24	28.0				
Non-SCC	24	25.1				
Pretreatment CEA			0.076	0.67	0.45–0.99	0.047
< 5 ng/ml	27	30.9				
≥ 5 ng/ml	23	18.3				
Radiotherapy technique			0.128	1.09	0.66–1.79	0.743
3D-CRT	18	16.2				
IMRT	25	30.1				
Radiotherapy dose			0.014	0.66	0.42–1.04	0.071
≥ 60 Gy	25	29.5				
< 60 Gy	19	14.9				
Concurrent chemotherapy						
EP	27	29.8	0.365			
PC	19	24.0				
Others	25	-				
Treatment modality			.012	0.61	0.42–0.88	0.008
CRT + CCT	27	30.4				
CRT	16	22.5				
Response			0.035	0.62	0.40–0.97	0.036
CR + PR	24	29.7				
SD	21	10.6				

**Fig. 2:**
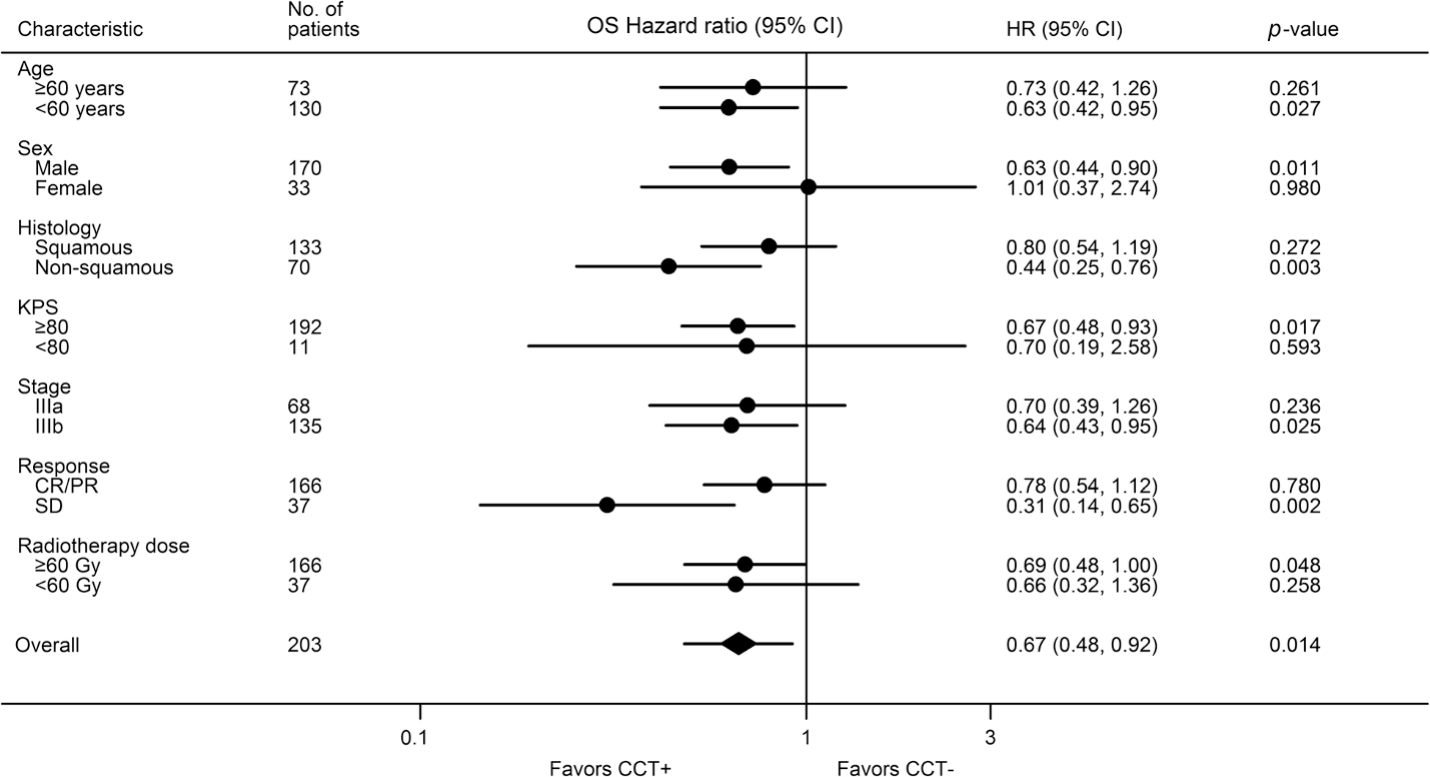
Hazard ratios of CCT to non-CCT in subgroup analysis according to study characteristics

In the subgroup analysis, the median OS and 5-year OS for patients receiving ≥2 cycles of CCT (27 months and 31.8 %) were better than those administered with <2 cycles of CCT (22 months and 18.7 %) (*p* = 0.317). The median time interval between completion of CRT to CCT was 6 weeks. The median OS and 5-year OS for patients with intervals ≤ 6 weeks (28 months and 34.4 %) were not statistically different from those with intervals > 6 weeks (25 months and 24 %) (*p* = 0.281). A forest plot of HRs for OS stratified by study characteristics is shown in Fig. 2. The survival advantages of CCT were statistically significant for males (HR: 0.63; 95 % CI, 0.44–0.90; *p* = 0.011), patients with age < 60 years (HR: 0.63; 95 % CI, 0.42–0.95; *p* = 0.027), non-squamous histology (HR: 0.44; 95 % CI, 0.25–0.76; *p* = 0.003), pretreatment KPS ≥ 80 (HR: 0.67; 95 % CI, 0.48–0.93; *p* = 0.017), stage IIIb (HR: 0.64; 95 % CI, 0.43–0.95; *p* = 0.025), SD (HR: 0.31; 95 % CI, 0.14–0.65; *p* = 0.002) and radiotherapy dose ≥ 60 Gy (HR: 0.69; 95 % CI, 0.48 − 1.00; *p* = 0.048).

### Toxicity

The treatment-related acute toxicities during the CRT and the CCT phase are listed in Table 3. The incidence of grade ≥ 3 hematological toxicities between the CCT group and the non-CCT group was similar (30.1 % vs. 34.4 %; *p* = 0.509) during the CRT phase. In patients receiving CCT, 15 % experienced grade ≥ 3 hematological toxicities during the CCT phase and no patient had grade 5 hematological toxicities. The incidence of grade ≥ 3 esophagitis was comparable between the CCT group and the non-CCT group during the CRT phase (9.7 % vs. 13.3 %; *p* = 0.422). Grade ≥ 3 radiation pneumonitis occurred at similar rates between the CCT and the non-CCT group during the CRT (0.0 % vs. 2.2 %; *p* = 0.195) and CCT phase (5.3 % vs. 3.3 %; *p* = 0.737). A total of 4 patients died of grade 5 radiation pneumonitis, including 2 (2.2 %) in the CCT group and 2 in the non-CCT (1.8 %) group.

**Table 3:** Treatment-related toxicities

Toxicity	CRT phase				CCT phase			
	CCT (%)	Non-CCT (%)	Total	*p*-value	CCT (%)	Non-CCT (%)	Total	*p*-value
Hematological				0.509				-
Grade 3/4	34 (30.1)	31 (34.4)	65 (32.0)		17 (15.0)	-	-	
Grade 1/2	79 (69.9)	59 (65.6)	138 (68.0)		96 (85.0)	-	-	
Esophagitis				0.422				-
Grade 3	11 (9.7)	12 (13.3)	23 (11.3)		-	-	-	
Grade 1/2	102 (90.3)	78 (86.7)	180 (88.7)		-	-	-	
Radiation pneumonitis				0.195				
Grade ≥ 3	0 (0.0)	2 (2.2)	2 (1.0)		6 (5.3)	3 (3.3)	9 (4.4)	0.737
Grade 1/2	113 (100.0)	88 (97.8)	201 (99.0)		107 (94.7)	87 (96.7)	194 (95.6)	

## Discussion

The outcomes of LA-NSCLC are relatively poor, with a high possibility of residual disease after definitive CRT. Thus, many clinical trials have investigated the role of additional CCT. To date, three randomized phase III studies [6, 9, 10] have been carried out to explore the efficacy and toxicity of CCT, among which only one has been published as a full article. HOG [6] reported that consolidation docetaxel yielded no survival benefit (median OS, 21.2 months vs. 23.2 months; *p* = 0.883) with an increased risk for grade 3/4 pneumonitis (9.6 % vs. 1.4 %; *p* < 0.001), infections (11 % vs. 0 %; *p* = 0.003), hospitalization (28.8 % vs. 8.1 %) and treatment-related death (5.5 % vs. 0 %; *p* = 0.058). In the GILT [9] study, consolidation oral vinorelbine (NVBo) and cisplatin (P) after NVBo plus P failed to prolong the median PFS (6.4 months vs. 5.5 months; *p* = 0.630) and 4-year OS (25.3 % vs. 21.4 %). The multinational CCheIN trial [10] reported that consolidation DP (docetaxel plus cisplatin) after concurrent weekly DP resulted in a PFS (median PFS, 9.1 months vs. 8.1 months; *p* = 0.390) and a OS (median OS, 21.8 months vs. 20.6 months; *p* = 0.490) that were similar to those of the observation group. A recently reported pooled-analysis including forty-one phase II/III studies with 3479 patients also failed to provide significant survival benefit of CCT for LA-NSCLC. Unlike HOG, the GILT study and CCheIN trial observed that the addition of CCT did not increase the toxicities. Despite the negative results mentioned above, many oncologists still attempt to deliver CCT for LA-NSCLC patients with good performance status after CRT in routine clinical practice, at least partially due to a poor survival rate of less than 20 % at 5 years and a significant survival benefit achieved by CCT in stage IV disease.

The long-term results of this retrospective study suggest that CCT further prolongs survival compared with CRT alone for LA-NSCLC without increased toxicities. Although more patients in the CCT group had a positive selection factors (female, younger age and a lighter history of smoking), the multivariate analysis was able to account for those selection bias and showed that CCT was a positive prognostic factor for OS and PFS. For patients in the CCT group, the encouraging median OS and 5-year OS were 27 months and 30.4 %, respectively, which were superior to those reported in randomized clinical trials [6, 9, 10] and comparable to the survival results in SWOG 9504. The median OS and 5-year OS were 16 months and 22.5 %, respectively, in the non-CCT group, which were similar to the historical controls [4, 7]. Although there was no difference regarding LRPFS or DMFS between the CCT group and the non-CCT group, CCT prolonged survival compared with CRT alone, which may be attributed to several reasons as follows. First, the multivariate analysis for PFS showed that CCT was an independent favorable prognostic factor (HR = 0.643; 95 % CI, 0.441–0.937; *p* = 0.022), though we found that the LRPFS (*p* = 0.265) and DMFS (*p* = 0.779) outcomes were similar between the CCT and non-CCT group. The improvement in disease control may translate into improved survival. The improvement in disease control may translate into improved survival. The multivariate analysis for PFS showed that CCT was an independent favorable prognostic factor (HR = 0.643; 95 % CI, 0.441–0.937; *p* = 0.022). A second explanation is that ethnicity may affect the efficacy of CCT. Our result is consistent with a recent pooled analysis [2] that suggested that survival was better in Asian patients when CCT was delivered, though this improvement was not statistically significant. Soo et al.[11] reported that the survival and response rate to chemotherapy were better in Asian patients with lung cancer, while the treatment-related toxicities were more severe than in Caucasian patients. To date, the exact mechanisms with which ethnicity affects the efficacy of CCT are unknown. The interethnic difference may be attributable to differences in the genetic backgrounds or environment and culture. Third, it should be noted that the actually delivered cycles of CCT in most studies were relatively lower (0.7 to 3.1, average: 1.5) than those observed in our study (the median number was 3 and 89.4 % of patients completed ≥2 cycles of CCT). Last, bias may be involved in such a retrospective study. The choice of oncologists and patients may influence the administration of CCT. Treatment compliance was higher in patients in the CCT group than in those in the non-CCT group because some patients refused CCT despite the oncologists’ suggestion. Treatment compliance could impact patients’ routine follow up and motivation for salvage treatment after progression, which influences the outcome. The reason why CCT resulted in no significant increase in toxicities may be increased use of IMRT (85.8 % vs. 71.1 %; *p* = 0.010) and timely management of toxicity, as IMRT may decrease esophageal and pulmonary toxicity compared with 3D-CRT by increasing target conformity [12, 13].

Our study also suggested that CCT may lead to significant OS benefit for males, patients with age < 60 years, non-squamous histology, pretreatment KPS ≥ 80, stage IIIb, SD and radiotherapy dose ≥ 60 Gy. It seems plausible that fit patients with higher risk of distant metastasis would benefit from CCT. Interestingly, the fact that the HR for patients achieving SD is favoring CCT, which is contrary to Jeremic [14] holding the view that patients with a CR or a PR rather than those with a SD were likely to benefit from CCT. However, the number of patients with SD in our study was too small to draw a conclusion.

Prognostic factors are essential to understand the disease process, select treatments and design clinical trials. Numerous studies have investigated the prognostic factors for LA-NSCLC with inconsistent results. The commonly recognized favorable prognostic factors include stage IIIa, good performance status, non-significant weight loss, and female gender [15–17]. In our study, the multivariate analyses identified pretreatment CEA ≥ 5 ng/ml, no CCT, and SD after CRT as predictive of worse OS. Age < 60 years, pretreatment CEA ≥ 5 ng/ml, and no CCT were significantly associated with poor PFS. Our study did not show a significant association between OS or PFS and the widely recognized prognostic factors mentioned above, which may be the result of a relatively small sample size and under-representation of patients with pretreatment KPS < 80 (5.4 %) and weight loss ≥ 5 % (18 %).

Similar to our results, a retrospective study [18] reported that the clinical tumor response was significantly associated with OS. Kim et al. [19] found a five-fold likelihood of long term survival for responders (CR or PR) compared to non-responders (SD or PD) (*p* = 0.067). Because the clinical tumor response can be assessed soon after CRT, this approach may aid in the following treatment decision according to clinical tumor response to initial CRT because non-responders may need more aggressive treatment.

The prognostic role of age for LA-NSCLC is contradictory. A Radiation Therapy Oncology Group (RTOG)-based analysis [17] found that age ≤ 70 years was associated with improved survival. Nevertheless, the secondary analysis of RTOG 9410 [20] demonstrated that in patients treated with CRT, the median OS was longer for patients aged ≥ 70 years (22.4 months vs. 15.5 months, *p*-value not provided). Numerous recent trials [21–23] suggested that CRT yielded similar treatment outcome for fit older patients compared with younger patients, which agreed with our results that the elderly (age ≥ 60 years) were non-inferior to the young (age < 60 years) with respect to OS. The reason why age < 60 years acted as a negative predictor for PFS is unknown. The difference in the biological behavior between younger and older patients warrants further investigation.

Although our study is based on a relatively large sample size with a long follow-up period, it has some limitations. Like all other retrospective studies, our study is inevitably subject to multiple biases. Moreover, the CCT regimens were largely heterogeneous, which hindered our study from further exploring the most effective CCT regimen.

## Conclusions

This retrospective study suggested that CCT further prolonged survival compared with CRT alone for LA-NSCLC without increasing treatment-related toxicities. Subgroup analysis identified that the survival advantages of CCT were more significant for males, patients with age < 60 years, non-squamous histology, pretreatment KPS ≥ 80, stage IIIb, SD and radiotherapy dose ≥ 60 Gy. Further prospective investigations that incorporate patient characteristics and treatment response are needed to validate our results.

## Additional file

Additional file 1: Table S1. Results of the univariate and multivariate analyses of prognostic factors for PFS. (DOCX 20 kb)

## Abbreviations

3D-CRT: Three-dimensional conformal radiotherapy
AJCC: American joint committee on cancer
CBC: Complete blood cell count
CCT: Consolidation chemotherapy
CEA: Carcinoembryonic antigen
CI: Confidence interval
CR: Complete response
CRT: Concurrent chemoradiotherapy
CSS: Cancer specific survival
CTCAE: Common terminology criteria for adverse events
DMFS: Distant metastasis-free survival
DP: Docetaxel plus cisplatin
EP: Etoposide plus cisplatin
HOG: Hoosier oncology group
IMRT: Intensity modulated radiotherapy
KPS: Karnofsky performance score
LA-NSCLC: Locally advanced non-small-cell lung cancer
LRPFS: Local regional progression-free survival
NVBo: Oral vinorelbine
OS: Overall survival
P: Cisplatin
PC: Paclitaxel plus carboplatin
PET: Positron emission tomography
PFS: Progression-free survival
PR: Partial response
RECIST: Response evaluation criteria for solid tumors
RTOG: Radiation therapy oncology group
SCC: Squamous cell carcinoma
SD: Stable disease
SWOG: Southwest oncology group
