# Supplementary material for: Consolidation chemotherapy may improve survival for patients with locally advanced non-small-cell lung cancer receiving concurrent chemoradiotherapy - retrospective analysis of 203 cases
Source: BMC Cancer. 2015 Oct 16;15:715. doi: 10.1186/s12885-015-1710-2 (PMC4609086; doi:10.1186/s12885-015-1710-2)
Supplement: Additional file 1: Table S1. — Results of the univariate and multivariate analyses of prognostic factors for PFS. (DOCX 20 kb) [file 12885_2015_1710_MOESM1_ESM.docx]

**Additional file 1**. Results of the univariate and multivariate analyses of prognostic factors for PFS

|  | **Univariate analysis** | | |  | **Multivariate analysis** | | |
| --- | --- | --- | --- | --- | --- | --- | --- |
| **Characteristic** | **MST (mos)** | **5-yr PFS (%)** | ***p*-value** |  | **HR** | **95% CI** | ***p*-value** |
| Gender |  |  | 0.252 |  | 1.01 | 0.60-1.70 | 0.969 |
| Male | 11 | 23.7 |  |  |  |  |  |
| Female | 11 | 9.8 |  |  |  |  |  |
| Age |  |  | 0.095 |  | 0.60 | 0.40-0.89 | 0.012 |
| <60 years | 11 | 17.1 |  |  |  |  |  |
| ≥60 years | 13 | 29.5 |  |  |  |  |  |
| Weight loss |  |  | 0.170 |  | 1.51 | 0.87-2.62 | 0.139 |
| <5% | 11 | 20.5 |  |  |  |  |  |
| ≥5% | 16 | 27.1 |  |  |  |  |  |
| Smoking index |  |  | 0.398 |  |  |  |  |
| ≤400 | 11 | 17.4 |  |  |  |  |  |
| >400 | 12 | 24.2 |  |  |  |  |  |
| Pretreatment hemoglobin |  |  | 0.879 |  |  |  |  |
| <120 g/L | 16 | - |  |  |  |  |  |
| ≥120 g/L | 11 | 21.5 |  |  |  |  |  |
| Pretreatment KPS |  |  | 0.142 |  | 0.64 | 0.29-1.40 | 0.259 |
| <80 | 11 | 0 |  |  |  |  |  |
| ≥80 | 11 | 22.4 |  |  |  |  |  |
| Stage |  |  | 0.244 |  | 0.87 | 0.57-1.33 | 0.522 |
| IIIa | 13 | 27.7 |  |  |  |  |  |
| IIIb | 11 | 18.2 |  |  |  |  |  |
| Histology subtype |  |  | 0.013 |  | 0.90 | 0.59-1.37 | 0.625 |
| SCC | 13 | 26.4 |  |  |  |  |  |
| Non-SCC | 10 | 12.4 |  |  |  |  |  |
| Pretreatment CEA |  |  | 0.000 |  | 0.48 | 0.33-0.70 | 0.000 |
| <5 ng/ml | 13 | 30.2 |  |  |  |  |  |
| ≥5 ng/ml | 7 | 9.6 |  |  |  |  |  |
| Radiotherapy technique |  |  | 0.974 |  |  |  |  |
| 3D-CRT | 12 | 13.8 |  |  |  |  |  |
| IMRT | 11 | 23.2 |  |  |  |  |  |
| Radiotherapy dose |  |  | 0.019 |  | 0.73 | 0.46-1.15 | 0.170 |
| ≥60 Gy | 12 | 24.2 |  |  |  |  |  |
| <60 Gy | 10 | 8.1 |  |  |  |  |  |
| Concurrent chemotherapy |  |  | 0.351 |  |  |  |  |
| EP | 13 | 26.9 |  |  |  |  |  |
| PC | 10 | 19.3 |  |  |  |  |  |
| Others | 11 | - |  |  |  |  |  |
| Treatment modality |  |  | 0.291 |  | 0.64 | 0.44-0.94 | 0.022 |
| CRT+CCT | 12 | 21.8 |  |  |  |  |  |
| CRT | 9 | 21.4 |  |  |  |  |  |
| Response |  |  | 0.049 |  | 0.72 | 0.45-1.14 | 0.162 |
| CR+PR | 12 | 23.1 |  |  |  |  |  |
| SD | 7 | - |  |  |  |  |  |
